# Effects of a Single Session of Active Release Technique on Gastrocnemius Delayed-Onset Muscle Soreness in Male Amateur Football Athletes: A Randomized Split-Body Pilot Study

**DOI:** 10.3390/healthcare14111581

**Published:** 2026-06-04

**Authors:** Theofanis Dimoulias, George Krekoukias, Maria Moutzouri, George Plakoutsis, Eleftherios Paraskevopoulos, Maria Papandreou

**Affiliations:** 1 Laboratory of Advanced Physiotherapy, Department of Physiotherapy, University of West Attica, 12243 Athens, Greece; gkrekoukias@uniwa.gr (G.K.); moutzouri@uniwa.gr (M.M.); gplakoutsis@uniwa.gr (G.P.); mpapand@uniwa.gr (M.P.); 2 School of Physical Education and Sports Science Department, National and Kapodistrian University of Athens, 17237 Dafne, Greece; elparaskev@phed.uoa.gr

**Keywords:** delayed-onset muscle soreness, gastrocnemius muscle, football athletes, active release technique, myofascial therapy

## Abstract

**Background/Objectives:** Delayed-Onset Muscle Soreness (DOMS) is a common consequence of eccentric exercise, characterized by pain, stiffness, and reduced function. This pilot study aimed to evaluate the effect of a single session of Active Release Technique (ART) on gastrocnemius DOMS in amateur football athletes. **Methods:** Twelve football athletes participated in a randomized split-body design study. All participants completed a bilateral eccentric heel-raise protocol to induce DOMS. One lower limb from each participant was randomly assigned to the ART group (single 8–10 min session), while the contralateral limb served as a control. Pain intensity was assessed using the Numeric Rating Scale (NRS) and the pressure pain threshold (PPT) was measured at baseline and at 24, 48, and 72 h post-exercise. **Results:** The ART group demonstrated significantly lower NRS scores at 24 h compared with the control group (*p* = 0.007, r = 0.77). At 48 and 72 h, NRS scores remained significantly lower (48 h: *p* = 0.001, d = 1.26; 72 h: *p* = 0.003, d = 1.11). PPT values were also significantly higher in the ART group at 24, 48 and 72 h (24 h: *p* = 0.026, d = 0.73; 48 h: *p* < 0.001, d = 1.42; 72 h: *p* = 0.006, d = 0.98). **Conclusions:** A single ART session may reduce pain and increase the pressure pain threshold following gastrocnemius DOMS in amateur football athletes. However, given the pilot design, small sample size, and absence of functional outcome measures, these findings should be interpreted with caution and confirmed in future studies.

## 1. Introduction

Delayed-Onset Muscle Soreness (DOMS) is a common consequence of unaccustomed or strenuous eccentric exercise and is characterized by muscle pain, stiffness, tenderness, and temporary reductions in functional performance [1]. The condition is believed to result from exercise-induced microtrauma to muscle fibers and connective tissue, followed by inflammatory responses, edema formation, and sensitization of group III and IV nociceptors, which collectively contribute to pain perception and mechanical hyperalgesia [1,2,3]. These pathophysiological mechanisms may alter neuromuscular function and movement patterns, potentially increasing injury risk and impairing athletic performance. Common symptoms include pain, stiffness, and pressure tenderness, leading to reduced force production and functional limitations associated with myofibrillar microtrauma [2]. These symptoms typically peak 24–48 h post-exercise and gradually subside within approximately 72 h [2,3].

DOMS is prevalent in both individual and team sports, including football, a sport associated with a high incidence of lower-limb muscle injuries [4]. In professional football, muscle strains account for more than 31% of all injuries. The physical demands of the sport, including accelerations, decelerations, sprinting, and rapid changes in direction, place substantial stress on the musculotendinous system and increase the likelihood of DOMS development [5,6]. In particular, gastrocnemius DOMS is common because of the explosive nature of football-specific movements such as jumping and kicking [5].

Due to its pivotal role in propulsion during running and sprinting, the gastrocnemius muscle is subjected to repetitive eccentric loading, making it particularly susceptible to strains and DOMS [7,8]. Gastrocnemius DOMS may impair muscular performance and induce compensatory movement patterns, thereby increasing biomechanical stress on the lower limbs of football athletes [1].

Given the impact of DOMS on athletic performance and recovery, several interventions have been proposed to reduce pain and accelerate recovery. Various techniques, including cryotherapy, active recovery, cold-water immersion, laser therapy, and acupuncture, have been investigated, with mixed results reported across studies [9,10,11]. Cryotherapy and cold-water immersion have been shown to reduce pain and muscle stiffness following eccentric exercise [9,12]. Active recovery strategies appear to be more effective than static stretching for post-match recovery in young football players [10]. Other modalities, including laser therapy and acupuncture, have also demonstrated potential analgesic effects; however, findings remain inconsistent [11].

Self-myofascial release techniques, such as foam rolling, have also been shown to reduce pain and improve mobility, although findings remain inconsistent [13,14,15,16]. Among football athletes, the effectiveness of foam rolling for the management of DOMS remains inconclusive [17]. Other soft-tissue techniques, such as instrument-assisted soft-tissue mobilization (IASTM), have demonstrated reductions in pain and increases in the pressure pain threshold; however, variability in treatment protocols limits the generalizability of these findings [18,19].

The existing literature is characterized by small sample sizes and methodological heterogeneity, with limited evidence specifically related to athletic populations [13,20]. Furthermore, research specifically addressing gastrocnemius DOMS in football players remains limited, as does evidence regarding interventions that facilitate a rapid and pain-free return to sport.

The Active Release Technique (ART) is a manual soft-tissue intervention designed to identify and treat myofascial adhesions and mobility restrictions through the application of precisely directed tension combined with active patient movement [21]. Originally developed by chiropractor P. Michael Leahy, the ART was conceptualized to address soft-tissue fibrosis and adhesions associated with repetitive strain, trauma, and overuse. Proposed or hypothesized therapeutic mechanisms of the ART may include improvements in tissue extensibility and fascial gliding, reductions in nociceptive input, and modulation of neuromuscular function [22]. However, these mechanisms remain largely theoretical, and current evidence supporting the ART is derived primarily from clinical and preliminary studies rather than direct mechanistic investigations.

Although the ART has been increasingly used in the management of musculoskeletal pain and overuse conditions, the current evidence supporting its effectiveness remains limited and methodologically heterogeneous. Existing studies are predominantly based on pilot trials, quasi-experimental designs, or case-based reports, often involving small sample sizes and lacking sham-controlled or blinded methodologies, thereby increasing the risk of performance and detection bias [22,23,24]. Nevertheless, preliminary findings have suggested potential short-term improvements in pain sensitivity and function following ART interventions. For example, a single ART session was associated with an increase in the pressure pain threshold in ice hockey athletes with adductor strains [23], while reductions in pain and improvements in function have been reported in recreational runners with gastrocnemius tightness [24]. However, to date, no studies have specifically investigated the effects of the ART on gastrocnemius DOMS in football athletes.

Based on preliminary evidence suggesting manual therapy interventions for DOMS-related pain modulation, it was hypothesized that ART-treated limbs would demonstrate lower subjective pain intensity (NRS) and higher pressure pain threshold (PPT) values compared with control limbs at 24, 48, and 72 h following DOMS induction. Given the pilot nature of the study, these hypotheses were considered exploratory, and the primary aim of the study was to assess feasibility and generate preliminary effect estimates to inform future adequately powered trials.

## 2. Materials and Methods

### 2.1. Participants

Twelve healthy adult male amateur football athletes were recruited from football teams in Attica. Eligibility criteria included an age of 18–40 years. Participants were required to have at least 3 years of continuous competitive experience, train at least 4 times per week (90 min/session), and participate in a minimum of 10 matches annually. All athletes refrained from training throughout the 4-day study protocol, completed their last training session at least 24 h before baseline assessment, and consumed their last meal at least 3 h prior to testing.

Inclusion criteria consisted of active male football athletes aged 18–40 years [25], a minimum of 3 years of football training experience, participation in at least 4 weekly training sessions, absence of lower-limb musculoskeletal injuries within the previous 3 months [26], and the ability to refrain from training during the study period.

Exclusion criteria included lower-limb surgery within the previous 6 months, current or recent musculoskeletal disorders, and ongoing physiotherapy treatment. Participants were also excluded if they self-reported a history of cardiovascular, respiratory, metabolic, neurological, autoimmune, or infectious diseases, or the use of medications that could potentially affect performance, recovery, or pain perception [27,28,29,30,31,32,33,34]. Eligibility was determined through a standardized health-history questionnaire and participant self-report prior to enrollment. No participants meeting the exclusion criteria were enrolled in the study.

### 2.2. Procedures

This pilot trial used an experimental split-body design involving healthy adult male amateur football athletes. In each participant, one lower limb was randomly assigned to the intervention group (Active Release Technique—ART), while the contralateral limb served as the control (No Treatment—NT).

Limb allocation was determined using a simple randomization procedure and concealed through sealed, opaque, sequentially numbered envelopes prepared by an independent researcher. The simple randomization (drawing lots) procedure was performed by the main researcher who was not involved in data collection or outcome assessment. Consistent with the split-body design, statistical analysis accounted for within-participant pairing, and comparisons between conditions were performed using paired statistical tests.

The study was conducted at the Laboratory of Advanced Physiotherapy, University of West Attica (UNIWA). All participants provided written informed consent prior to participation. The study protocol adhered to the principles of the Declaration of Helsinki and was approved by the UNIWA Ethics Committee (Protocol No. 16175/18 Febuary 2025). This pilot study was not prospectively registered (e.g., ClinicalTrials.gov), which is acknowledged as a study limitation.

As a pilot study, the primary objectives were to assess protocol feasibility and generate preliminary effect estimates to inform future larger-scale trials [35,36,37]. A dedicated feasibility assessment was conducted to evaluate the study procedures. Feasibility outcomes included recruitment rate, participant adherence, retention/dropout, and adverse events.

Twelve healthy adult male amateur football athletes were screened over the recruitment period, and all met the eligibility criteria, corresponding to a recruitment rate of 100%. All participants completed the intervention session and outcome assessments, resulting in full adherence to the study protocol. Participant retention was also 100%, with no withdrawals recorded before study completion. In addition, no adverse events related to the intervention were observed during the study period. Overall, these findings support the feasibility and acceptability of conducting a larger-scale trial using the current study procedures.

### 2.3. Outcome Measures

#### 2.3.1. Numeric Rating Scale (NRS)

Two outcome measures were assessed at baseline (current pain at rest) and at 24, 48, and 72 h following DOMS induction, reflecting the worst pain experienced during each corresponding time period. Subjective pain intensity was evaluated using the Numeric Rating Scale (NRS; 0 = “no pain”, 10 = “worst imaginable pain”) [38,39], a valid and reliable instrument for the assessment of DOMS [40]. Standardized instructions were provided consistently to all participants to ensure uniformity in pain reporting throughout the study period.

#### 2.3.2. Pressure Pain Threshold (PPT)

**Figure 1:**
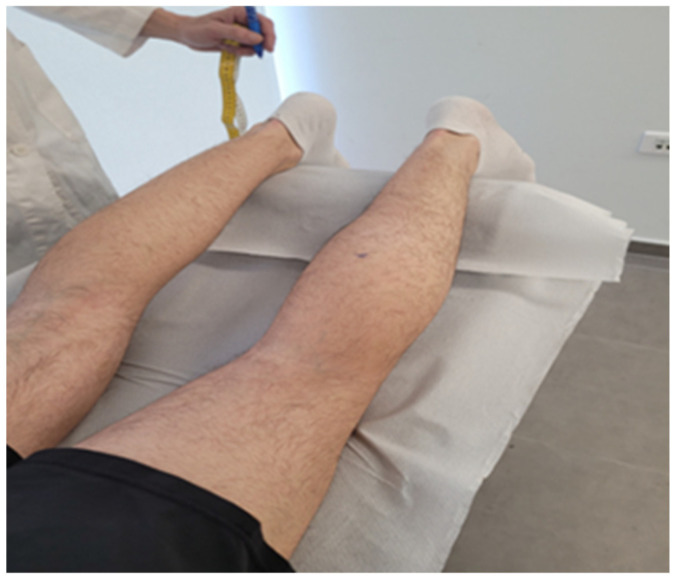
Identification and marking of the algometry measurement site, located at the midpoint between the popliteal crease and the level of the malleoli. Note: Participants provided written consent for the publication of identifiable photographs.

Objective pain was quantified using the pressure pain threshold (PPT), measured in kgf/cm^2^, with a calibrated digital algometer equipped with a 1 cm^2^ rubber probe, following established procedures [41,42]. The PPT was defined as the minimum amount of pressure required to elicit the first sensation of pain. Measurements were performed with participants in the prone position. A bolster was placed under the ankle to maintain knee flexion, and the knee angle was standardized at 30° using a universal manual goniometer to ensure consistency across assessments. The assessment site was standardized at the midpoint between the popliteal crease and the malleoli (Figure 1). Pressure was applied perpendicularly at a constant rate of 100 g/s, controlled using a metronome. Three measurements were obtained with 30 s rest intervals, and the mean value was used for analysis [43]. The algometer was zeroed and calibrated using objects of known mass prior to each testing session [41,44].

### 2.4. Intervention Protocol

All procedures, including administration of the ART intervention and outcome assessments, were performed by the same experienced physiotherapist, who had more than 3 years of clinical experience in musculoskeletal and sports rehabilitation. Owing to the nature of the intervention, assessor blinding was not feasible.

#### 2.4.1. DOMS Induction Protocol

**Figure 2:**
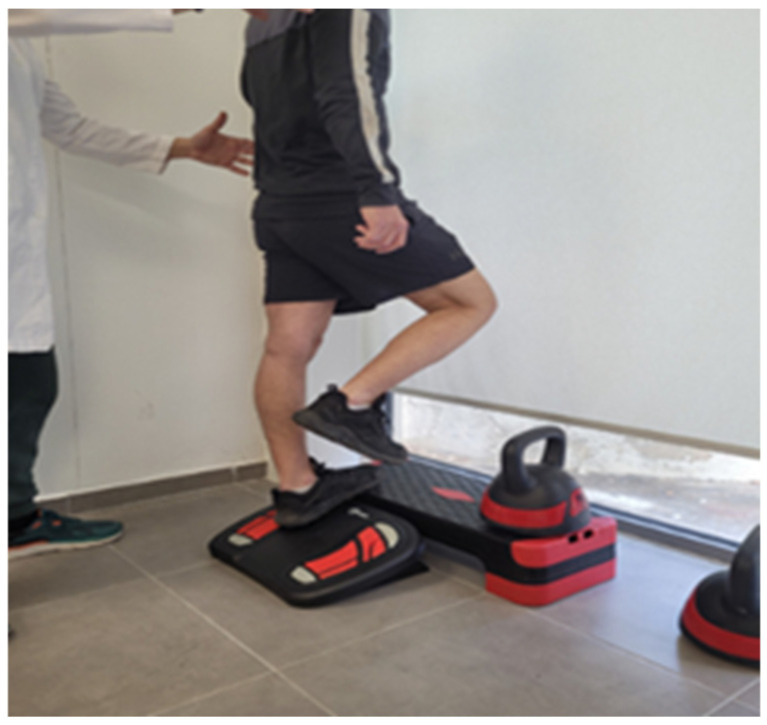
DOMS induction protocol: eccentric phase (heel lowering) [45]. Note: Participants provided written consent for the publication of identifiable photographs.

DOMS was induced using a standardized eccentric heel-raise protocol previously validated through MRI findings and biochemical markers [45]. Following a warm-up consisting of 2 sets of 15 repetitions per limb, participants performed 4 sets of 50 repetitions per limb on a 15 cm step platform positioned at a 35° incline to maximize mechanical loading. Athletes wore a weighted vest corresponding to approximately 40% of their body mass. Each repetition consisted of a 1 s concentric phase followed by a 3 s eccentric phase, paced at 60 bpm, with 60 s rest intervals between sets (Figure 2). A fifth set was performed to volitional exhaustion, defined as the inability to complete an additional repetition while maintaining proper technique. The total protocol duration was approximately 40–45 min. No adverse events (e.g., muscle strains or other complaints) were observed following DOMS induction. In addition, no stretching or recovery interventions were permitted during the study period.

#### 2.4.2. Active Release Technique (ART)

**Figure 3:**
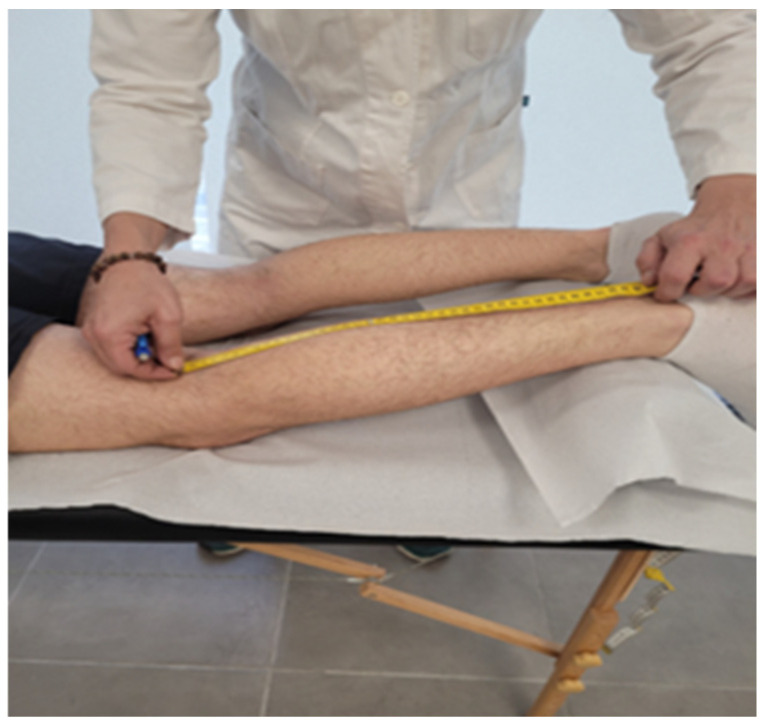
Identification and marking of the three standardized anatomical regions for Active Release Technique (ART) application on the gastrocnemius muscle. Note: Participants provided written consent for the publication of identifiable photographs.

**Figure 4:**
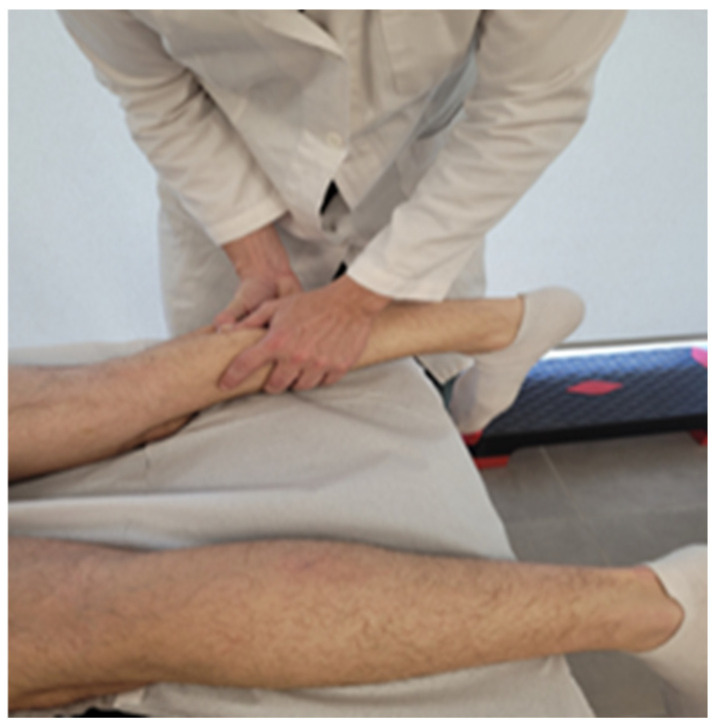
Application of the Active Release Technique (ART) to the gastrocnemius muscle. Pressure is applied to the point of tissue tension while the athlete is instructed to “pull the toes of the foot toward the head,” performing active ankle dorsiflexion from a position of full knee extension [24]. Note: Participants provided written consent for the publication of identifiable photographs.

The ART was administered in a single session immediately following completion of the DOMS induction protocol [23,24,45]. The randomized limb received the ART intervention, while the contralateral limb served as an untreated control. The technique was applied to 3 standardized anatomical regions of the gastrocnemius muscle (3 cm distal to the popliteal crease, the midpoint of the muscle belly, and 3 cm proximal to the Achilles tendon insertion) to ensure consistency across participants (Figure 3). Within each region, the final treatment point was identified through palpation based on the presence of locally increased tissue tension, in accordance with established ART principles [46,47]. Sustained manual pressure was applied using reinforced thumbs (one thumb placed over the other) to ensure stable and deep tissue contact while participants performed active knee extension and ankle dorsiflexion (Figure 4). This was followed by a brief passive stretch at the end range of motion. Two repetitions of 15–20 s each were performed per region, with 30 s rest intervals between applications. The total duration of the ART session was approximately 8–10 min.

Baseline measurements were obtained prior to the DOMS-inducing exercise protocol, while the ART was administered immediately following completion of the DOMS induction procedure. Subsequent assessments were conducted at 24, 48, and 72 h after the intervention, with timing calculated from the end of the ART session.

### 2.5. Statistical Analysis

Statistical analysis was performed using SPSS version 29 (Statistical Package for the Social Sciences, IBM inc., Chicago, IL, USA). Data normality was assessed using the Shapiro–Wilk test separately for each condition (intervention group: ART; control group: NT) and at all time points (baseline, 24, 48 and 72 h) (Appendix A; Table A1). Variables demonstrating normal distribution were analyzed using parametric methods, whereas non-normally distributed variables were analyzed using non-parametric methods. No missing data were identified. Given the split-body design, statistical analyses accounted for the paired nature of the data. For normally distributed variables, paired-sample *t*-tests were used to compare ART and NT limbs at each time point, whereas the Wilcoxon signed-rank test was applied for non-normally distributed variables. In addition to *p*-values, effect sizes were calculated to quantify the magnitude of differences (Cohen’s d for parametric tests and r for non-parametric tests), and 95% confidence intervals (CIs) were reported where appropriate (Table 1 and Table 2). Statistical significance was set at *p* < 0.05.

## 3. Results

### 3.1. Participant Characteristics

Twelve healthy male amateur football athletes (n = 12) completed the study. Participants ranged in age from 19 to 27 years. In accordance with the split-body design, each participant contributed one limb to the ART group and the contralateral limb to the control group (n = 12 limbs per group). Baseline anthropometric and clinical characteristics of the participants are presented in Table 3.

### 3.2. Baseline Comparisons

At baseline, no significant differences between groups were observed using the Wilcoxon signed-rank test for either NRS scores (Median-ART = 0.0, Median-NT = 0.0, *p* = 0.317, *p* ≥ 0.05) or PPT values (Median-ART = 8.35, Median-NT = 9.25, *p* = 0.791, *p* ≥ 0.05).

### 3.3. 24 h Post-Exercise

**Figure 5:**
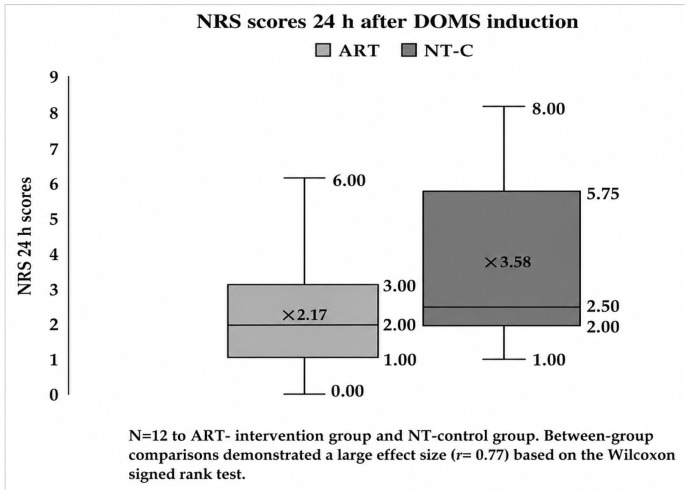
Box plot of the distribution of Subjective Pain Rating (NRS, 0–10) values at 24 h post-intervention, showing a statistically significant reduction in pain in the ART-intervention group compared to the NT-control group.

**Figure 6:**
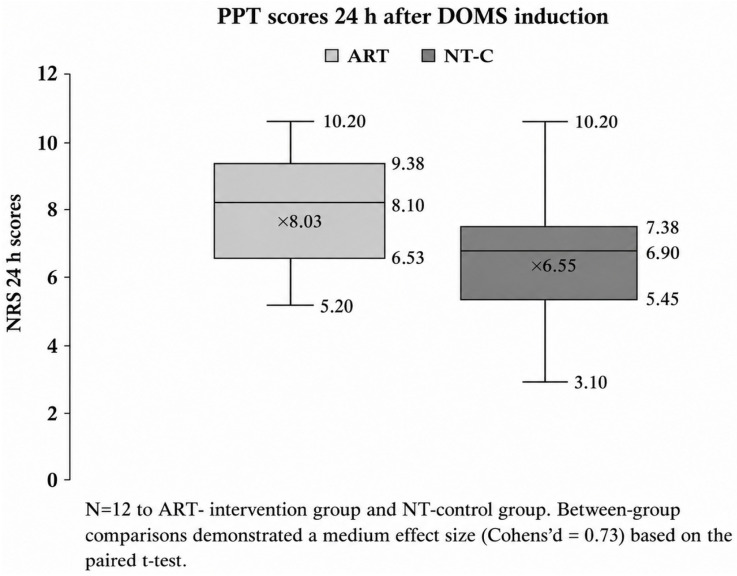
Box plot of the distribution of Objective Pain Assessment values (PPT, kgf/cm^2^) at 24 h post-intervention, demonstrating a statistically significant increase in pain tolerance in the ART-intervention group compared to the NT-control group.

At 24 h post-DOMS induction, the Wilcoxon signed-rank test revealed significant differences between groups for the NRS (Median-ART = 2.0, Median-NT = 2.5, *p* = 0.007, *p* ≤ 0.05). The ART group demonstrated lower NRS scores compared with the NT-control group (Figure 5). A significant difference was also observed for PPT values (*p* = 0.026), with higher PPT values in the ART condition (Figure 6).

### 3.4. 48 h Post-Exercise

**Figure 7:**
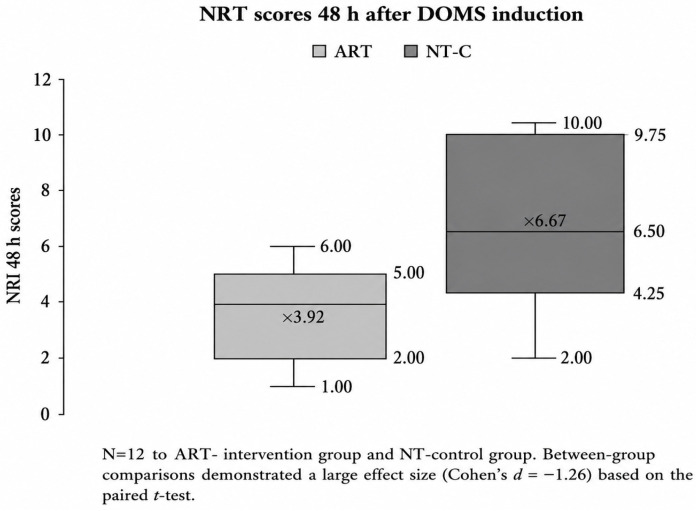
Box plot of the distribution of Subjective Pain Rating (NRS, 0–10) values at 48 h post-intervention, showing a statistically significant reduction in pain in the ART-intervention group compared to the NT-control group.

**Figure 8:**
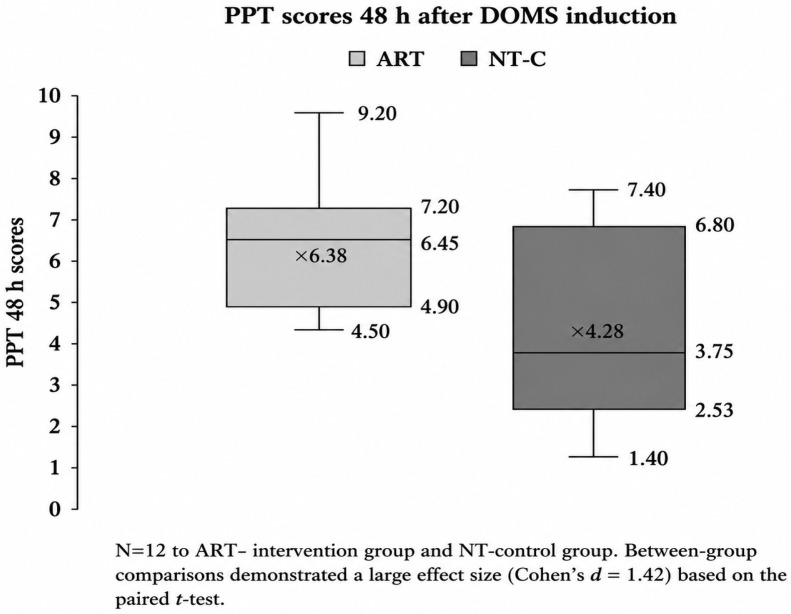
Box plot of the distribution of Objective Pain Assessment values (PPT, kgf/cm^2^) at 48 h post-intervention, demonstrating a statistically significant increase in pain tolerance in the ATR-intervention group compared to the NT-control group.

At 48 h post-exercise induction, significant differences were observed between groups. The ART group demonstrated lower NRS scores (3.92 ± 1.67) compared with the NT-control group (6.67 ± 2.80) (mean difference = −2.75 ± 2.17, t = −4.37, *p* = 0.001, *p* ≤ 0.05) (Figure 7). In addition, PPT values were significantly higher in the ART group (6.38 ± 1.95 kgf/cm^2^) compared with the control group (4.28 ± 2.14 kgf/cm^2^) (mean difference = 2.09 ± 1.47, t = 4.91, *p* < 0.001) (Figure 8).

### 3.5. 72 h of Post-Exercise

**Figure 9:**
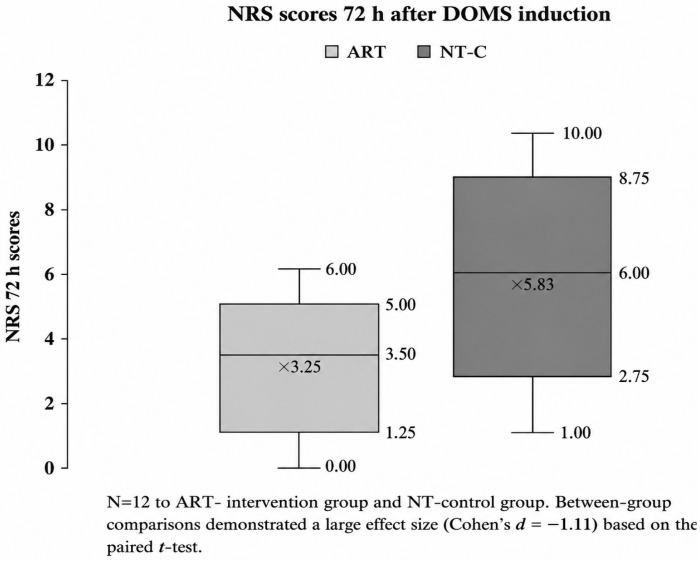
Box plot of the distribution of Subjective Pain Rating (NRS, 0–10) values at 72 h post-intervention, indicating a statistically significant reduction in pain in the ART-intervention group compared to the NT-control group.

**Figure 10:**
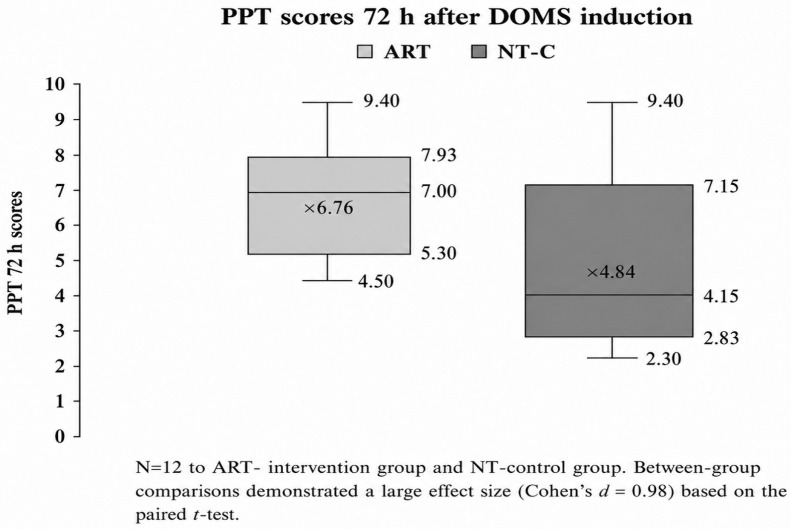
Box plot of the distribution of Objective Pain Assessment values (PPT, kgf/cm^2^) at 72 h post-intervention, demonstrating a statistically significant increase in pain tolerance in the ATR-intervention group compared to the NT-control group.

At 72 h post-exercise induction, significant differences were observed between groups. The ART group demonstrated lower NRS scores (3.25 ± 2.09) compared with the control group (5.83 ± 3.01) (mean difference = −2.58 ± 2.31, t = −3.86, *p* = 0.003, *p* ≤ 0.05) (Figure 9). Similarly, PPT values were significantly higher in the ART group (6.76 ± 1.55 kgf/cm^2^) compared with the control group (4.84 ± 2.37 kgf/cm^2^) (mean difference = 1.91 ± 1.93, t = 3.42, *p* = 0.006, *p* ≤ 0.05) (Figure 10).

## 4. Discussion

This pilot study estimated the preliminary effects of a single Active Release Technique (ART) session on gastrocnemius DOMS in amateur football athletes. ART significantly reduced perceived pain (NRS) at 24, 48 and 72 h and increased mechanical pressure tolerance (PPT) at 24, 48 and 72 h following DOMS induction.

At 24 h following DOMS induction, significant differences were observed in both subjective pain intensity (NRS) and pressure pain threshold (PPT), suggesting that the ART may influence both perceived pain and mechanical pain intensity during the early phase of DOMS. Although the effect size for the PPT at 24 h was moderate (d = 0.73), the finding indicates that measurable changes in mechanical pain sensitivity may occur earlier than previously assumed [9,12]. Similar improvements in pain-related outcomes during the first 24 h following eccentric exercise have been reported following interventions such as cryotherapy, cold-water immersion, foam rolling, and IASTM, although findings remain inconsistent across studies and athletic populations [11,17,18,48]. The observed reduction in NRS scores may additionally reflect the perceptual and affective dimensions of pain, which are influenced not only by peripheral nociceptive input but also by psychological, cognitive, and contextual factors [1,2,3]. Together, these findings suggest that ART may exert early hypoalgesic effects following DOMS induction; however, the underlying mechanisms remain unclear and require further investigation.

At 48 h—the typical peak of DOMS intensity—ART-treated limbs demonstrated significantly lower NRS scores and higher PPT values, indicating a potentially clinically meaningful reduction in pain sensitivity during peak soreness, although functional outcomes were not assessed. These findings are consistent with evidence suggesting that soft-tissue interventions may be more effective during the peak inflammatory phase [49,50]. Similar increases in the PPT following a single ART session have been reported in athletic populations, although methodological differences limit direct comparison [23]. These effects may be mediated through mechanoreceptor stimulation, including activation of Golgi tendon organs and Ruffini endings, potentially leading to autogenic inhibition, reduced muscle tension, improved local circulation, and sensory desensitization [51,52]. However, these proposed mechanisms remain hypothetical and have not yet been experimentally confirmed.

At 72 h, significant differences persisted for both outcomes, suggesting that a single 8 min ART session may contribute to an improved short-term recovery profile in athletes and physically active individuals. This prolonged effect may reflect continued reductions in muscle stiffness and pain sensitivity, potentially facilitating more efficient tissue recovery following exercise-induced muscle damage. However, the underlying physiological mechanisms remain unclear, and further studies with larger samples and longitudinal follow-up are required to determine the durability and clinical relevance of these effects. These findings are consistent with studies reporting benefits lasting up to 72 h following interventions such as cryosauna, cold-water immersion, photobiomodulation, and IASTM [11,12,18,53]. The ART may enhance microcirculation, reduce inflammatory by-products, and improve fascial gliding, thereby facilitating a faster restoration of tissue homeostasis [23,47,54].

### 4.1. Clinical Relevance

Football athletes are exposed to high eccentric demands, including rapid accelerations, decelerations, and frequent changes in direction, which increase the susceptibility of the gastrocnemius muscle to DOMS and subsequent declines in performance [5,6]. The present findings suggest that the ART may represent a potentially useful and time-efficient recovery approach for this population; however, its clinical relevance requires further investigation using functional outcome measures. The combined use of the NRS and PPT enabled a comprehensive assessment of both subjective and objective pain responses, while the brief 8 min application time allows ART to be integrated into training and competition schedules without the need for specialized equipment. The ART may therefore serve as a useful adjunct within multimodal recovery programs in football settings.

### 4.2. Comparison with Existing Literature

Research examining the effects of the ART on DOMS remains limited, and, to date, no previous study has specifically investigated gastrocnemius DOMS in football athletes. The present findings are consistent with earlier reports demonstrating short-term analgesic effects of the ART on pain sensitivity [22,23,24], although the broader evidence base regarding the ART remains limited and heterogeneous.

Systematic reviews investigating manual therapy interventions, including the ART, have generally concluded that although short-term improvements in pain and pressure pain sensitivity may occur, the overall certainty of evidence remains low due to small sample sizes, lack of blinding, and inconsistent methodologies [55]. Therefore, the current findings should be interpreted with caution. Nevertheless, the present study contributes to the emerging literature by investigating the ART within a sport-specific context, specifically gastrocnemius DOMS in football athletes, and by reporting improvements in both subjective pain intensity and pressure pain threshold during the 24–72 h recovery period.

## 5. Limitations and Future Directions

Several limitations of this pilot study should be acknowledged. First, the study was not prospectively registered (e.g., on ClinicalTrials.gov). Future studies investigating the same research objective should include prospective registration and recruit larger samples to ensure adequate statistical power. In addition, the sample was restricted to healthy adult male amateur football athletes, limiting the generalizability of the findings to female athletes, youth players, or elite-level populations. Although participants were instructed to maintain their usual daily habits, lifestyle factors such as nutrition, hydration, and sleep were not strictly controlled. Furthermore, limb dominance was not assessed, which may influence load distribution and muscle stress during eccentric exercise protocols [56,57]. This factor may have affected baseline muscle characteristics, as well as the physiological response to DOMS induction and the ART intervention. An additional limitation relates to the assumptions inherent to the split-body design. Although one limb served as an untreated control, the ART may induce systemic or centrally mediated hypoalgesic responses extending beyond the locally treated region. Consequently, potential crossover effects between limbs cannot be excluded. Another important limitation was the absence of a sham control group. Although the split-body design partially controlled for inter-individual variability, the inclusion of a sham intervention would have strengthened internal validity. In addition, the manual nature of the ART precluded assessor blinding. Future studies may therefore benefit from parallel-group or sham-controlled designs to better isolate local treatment effects. Finally, the absence of functional outcome measures (e.g., strength or range of motion assessment) limits the interpretation of potential functional implications beyond pain-related outcomes. Given the pilot nature and small sample size of the present study, paired statistical analyses were applied within the split-body design; however, future studies with larger samples should consider the use of mixed-effects models to more comprehensively account for within-participant correlations. In addition, no adjustment for multiple comparisons was applied across the repeated outcome assessments and time points. Consequently, the reported *p*-values should be interpreted with caution, as the risk of Type I error may be increased. Future adequately powered studies should consider multiplicity-adjusted analyses within a longitudinal modeling framework.

Future research should investigate the effects of ART in larger cohorts and more diverse athletic populations, including female athletes and youth players, while incorporating biomarkers such as creatine kinase and evaluating additional muscle groups. Furthermore, the inclusion of functional and performance-related outcomes—such as joint range of motion, muscle strength, and sport-specific performance tests—would help determine whether the observed reductions in pain translate into clinically meaningful improvements in athletic recovery and readiness. Finally, long-term follow-up assessments may provide insight into the persistence and potential clinical relevance of ART-related effects beyond the acute phase of muscle soreness.

## 6. Conclusions

In conclusion, this pilot study provides preliminary evidence that a single 8–10 min ART session may be associated with reduced subjective pain and increased mechanical pain thresholds in amateur football athletes experiencing gastrocnemius DOMS. Significant effects were observed at 24, 48, and 72 h for both NRS and PPT outcomes. These findings suggest that ART may have potential as a time-efficient adjunct within programs; however, its clinical significance and effects on functional recovery remain to be established in larger controlled studies.

However, given the pilot design, small sample size, and absence of functional outcome measures, these findings should be interpreted with caution. While the ART may have potential as a time-efficient adjunct within recovery programs, further adequately powered, sham-controlled studies are required to confirm its effectiveness and determine its clinical relevance.

From a practical perspective, these findings may be of interest to clinicians and sports practitioners involved in the management of exercise-induced muscle soreness. As a brief and non-invasive intervention, the ART may have potential as an adjunct within multimodal rehabilitation and recovery programs. However, further adequately powered and sham-controlled studies are required to determine its effectiveness, clinical relevance, optimal treatment protocols, and long-term effects across different athletic populations.

## Figures and Tables

**Table 1:** Effect sizes (Cohen’s d) and 95% confidence intervals for parametric paired comparisons between the ART and NT groups. Comparisons were analyzed using paired-sample *t*-tests following confirmation of normality using the Shapiro–Wilk test.

Groups Comparisons ART & NT	Cohens’ d	Effect Size	95% CI	
			Lower	Upper
NRS time 48 h	−1.26	Large	−4.13	−1.16
NRS time 72 h	−1.11	Large	−4.05	−1.11
PPT time 24 h	0.73	Medium	0.21	2.73
PPT time 48 h	1.42	Large	1.15	3.03
PPT time 72 h	0.98	Large	0.68	3.14

Note: All comparisons presented in this table were analyzed using paired-sample *t*-tests following confirmation of normality with the Shapiro–Wilk test (Appendix A; Table A1). Effect sizes are reported as Cohen’s d. Outcomes demonstrating non-normal distribution (NRS at baseline and 24 h) were analyzed separately using the Wilcoxon signed-rank test and are presented in Table 2.

**Table 2:** Effect sizes (r) for Wilcoxon signed-rank test comparisons between the ART and NT groups for outcomes demonstrating non-normal distribution at baseline and 24 h.

Groups Comparisons ART & NT	Z	Effect Size (r)
NRS time 0 h	0.00	0.00—Negligible
NRS time 24 h	−2.69	0.77—Large
PPT time 0 h	−0.26	0.28—Small

Note: Comparisons presented in this table demonstrated non-normal distribution according to the Shapiro–Wilk test (Appendix A; Table A1) and were analyzed using the Wilcoxon signed-rank test. Effect sizes are reported as r. Outcomes demonstrating normal distribution are presented in Table 1.

**Table 3:** Baseline characteristics of participants (Mean ± SD).

Variable	Participants (N = 12)
Age (years)	21.42 ± 2.52
Body weight (kg)	74.25 ± 9.20
Height (cm)	175.08 ± 3.90
BMI (kg/m^2^)	23.93 ± 2.34
Training experience (years)	4.33 ± 1.21

Note: Baseline characteristics are identical across groups because both limbs belong to the same participants in a within-subject (split-body) design.

## Data Availability

The data presented in this study are available on request from the corresponding author due to ethical and privacy restrictions.

## Appendix A

**Table A1:** Shapiro–Wilk results for assessing data normality for each variable for the intervention (ART) group and for the control (NT) group for the variables NRT (Numeric Rating Scale) and PPT (Pressure Pain Threshold) across all time points from 0 to 72 h.

Shapiro–Wilk	Statistic	Sign.	Normality
NRS time 0 (ART)	0.465	0.000	Not normal
NRS time 0 (NT)	0.552	0.000	Not normal
NRS time 24 (ART)	0.898	0.150	Normal
NRS time 24 (NT)	0.839	0.027	Not normal
NRS time 48 (ART)	0.811	0.013	Normal
NRS time 48 (NT)	0.927	0.354	Normal
NRS time 72 (ART)	0.897	0.144	Normal
NRS time 72 (NT)	0.928	0.363	Normal
PPT time 0 (ART)	0.845	0.032	Not normal
PPT time 0 (NT)	0.922	0.302	Normal
PPT time 24 (ART)	0.949	0.627	Normal
PPT time 24 (NT)	0.936	0.452	Normal
PPT time 48 (ART)	0.931	0.393	Normal
PPT time 48 (NT)	0.890	0.120	Normal
PPT time 72 (ART)	0.963	0.819	Normal
PPT time 72 (NT)	0.879	0.086	Normal
